# Trends in Hospital Costs and Levels of Services Provided for Children With Bronchiolitis Treated in Children’s Hospitals

**DOI:** 10.1001/jamanetworkopen.2021.29920

**Published:** 2021-10-26

**Authors:** Robert J. Willer, Eric R. Coon, Wade N. Harrison, Shawn L. Ralston

**Affiliations:** 1 Division of Pediatric Hospital Medicine, Department of Pediatrics, University of Utah School of Medicine, Primary Children’s Hospital, Salt Lake City; 2 Division of Pediatric Hospital Medicine, Department of Pediatrics, University of North Carolina at Chapel Hill, Chapel Hill; 3 Division of Pediatric Hospital Medicine, Department of Pediatrics, University of Washington, Seattle

## Abstract

**Importance:**

Increasing hospital costs for bronchiolitis have been associated with increasing patient complexity and mechanical ventilation. However, the associations of illness severity and diagnostic coding practices with bronchiolitis hospitalization costs have not been examined.

**Objective:**

To investigate the association of patient complexity, illness severity, and diagnostic coding practices with bronchiolitis hospitalization costs.

**Design, Setting, and Participants:**

This retrospective cross-sectional study included 385 883 infants aged 24 months or younger who were hospitalized with bronchiolitis at 39 hospitals in the Pediatric Health Information System database from January 1, 2010, to December 31, 2019.

**Exposure:**

Hospitalization for bronchiolitis.

**Main Outcomes and Measures:**

Inflation-adjusted standardized unit cost (expressed in dollar units) per hospitalization over time. A nested subgroup analysis was performed to further examine factors associated with changes in cost.

**Results:**

A total of 385 883 bronchiolitis hospitalizations were studied; the patients had a mean (SD) age of 7.5 (6.4) months and included 227 309 of 385 883 boys (58.9%) and 253 870 of 385 883 publicly insured patients (65.8%). Among patients hospitalized with bronchiolitis, the median standardized unit cost per hospitalization increased significantly during the study period (from $5636 [95% CI, $5558-$5714] in 2010 to $6973 [95% CI, $6915-$7030] in 2019; *P* < .001 for trend). Similar increases in cost were observed among subgroups of patients without a complex chronic condition and without the need for mechanical ventilation. However, costs for patients without a complex chronic condition or mechanical ventilation, who received care outside the intensive care unit did not change in an economically significant manner (from $4803 [95% CI, $4752-$4853] in 2010 to $4853 [95% CI, $4811-$4895] in 2019; *P* < .001 for trend), suggesting that intensive care unit use was a primary factor associated with cost increases. Substantial changes in coding practices were observed. Among patients hospitalized with bronchiolitis, 1.2% (95% CI, 1.1%-1.3%) were assigned an APR-DRG (All Patient Refined Diagnosis Related Group) for respiratory failure in 2010, which increased to 21.6% (95% CI, 21.2%-21.9%) in 2019 (*P* < .001 for trend). Increased costs and coding intensity were not accompanied by objective evidence of worsening illness severity.

**Conclusions and Relevance:**

This cross-sectional study suggests that hospitalized children with bronchiolitis are receiving costlier and more intensive care without objective evidence of increasing severity of illness. Changes in coding practices may complicate efforts to study trends in the use of health care resources using administrative data.

## Key Points

**Question:**

What factors are associated with increasing hospital costs for patients with bronchiolitis?

**Findings:**

In this cross-sectional study of 385 883 bronchiolitis hospitalizations, children hospitalized in later years received more costly and intensive care without objective evidence of increasing severity of illness. Substantial changes in coding practices were observed, with an increasing proportion of patients diagnosed with respiratory failure between 2010 and 2019.

**Meaning:**

This study suggests that future research on trends in bronchiolitis should account for changes in the patterns of diagnostic coding.

## Teaser

This cross-sectional study of more than 385 000 children hospitalized with bronchiolitis investigates the association of the complexity of a patient’s condition, illness severity, and diagnostic coding practices with bronchiolitis hospitalization costs.

## Introduction

Bronchiolitis is one of the most common pediatric diagnoses, accounting for more than 100 000 inpatient admissions per year at a direct cost of more than $700 million annually.^1,2,3^ According to an analysis of the Kids’ Inpatient Database, hospitalizations decreased during the past decade yet costs increased, a trend associated with increases in the proportion of patients with complex chronic conditions and the use of mechanical ventilation.^1,4^ These findings conflict with a recent analysis of the Pediatric Health Information Systems (PHIS) database, which found that costs per hospitalization were stable despite higher rates of noninvasive ventilation (NIV) and intensive care unit (ICU) use over time.^5^

These disparate findings are likely associated with several factors. First, although the Kids’ Inpatient Database is a nationally representative sample of pediatric admissions, it does not include observation stays, which now constitute up to one-third of pediatric hospitalizations.^6,7,8^ Therefore, the Kids’ Inpatient Database underestimates total hospital resource use and costs for bronchiolitis while simultaneously overestimating costs per hospitalization because children with complex chronic conditions are less likely to be placed in observation.^9,10,11^ Second, the trends in hospital costs observed in the PHIS database were sensitive to the strategy used to identify patients, specifically including patients with bronchiolitis only in the primary diagnosis position compared with including patients with bronchiolitis in any position.^5^ Finally, as value-based payment models are increasingly adopted, children’s hospitals are focusing on an accurate case-mix adjustment, and changes in diagnostic coding practices are likely ocurring.^12^ Therefore, it is unclear to what extent apparent trends in resource use are associated with patient-level factors as opposed to coding practices.

Thus, we sought to explore trends in bronchiolitis hospital resource use and diagnostic coding in a cohort of patients at children’s hospitals using the PHIS database. Our objective was to investigate the association of patient-level factors and diagnostic coding practices with bronchiolitis hospitalization costs.

## Methods

### Design and Data Source

This retrospective cohort included infants 24 months or younger hospitalized with bronchiolitis at 39 hospitals using the PHIS database from January 10, 2010, to December 31, 2019. The PHIS database is managed by the Children’s Hospital Association and includes patient-level data from children’s hospitals in the United States, including patient demographic characteristics, procedure codes, discharge diagnosis codes, and detailed billing information for laboratory, imaging, supply, and pharmacy charges. This study followed the Strengthening the Reporting of Observational Studies in Epidemiology (STROBE) reporting guideline and was determined to be non–human participant research by the University of Utah institutional review board and exempt from review.

### Study Population

Patients were included if they had an *International Classification of Diseases, Ninth Revision* (*ICD-9*) discharge diagnosis code for bronchiolitis (466.XX) in any position consistent with methods reported by Fujiogi et al.^1^ Two alternative methods of identifying bronchiolitis hospitalizations were also used in sensitivity analyses: (1) patients with a primary diagnosis of bronchiolitis and (2) patients assigned the All Patient Refined Diagnosis Related Group (APR-DRG) for bronchiolitis. For patients discharged after October 1, 2015 (the date of transition from *ICD-9* to the *International Statistical Classification of Diseases and Related Health Problems, Tenth Revision* [*ICD-10*]), a crosswalk available from the Centers for Medicare & Medicaid Services was used to convert *ICD-10* diagnosis codes to *ICD-9* diagnosis codes.^13^ Inpatient and observation status hospitalizations were included. Only hospitals with complete data available for each year from 2010 to 2019 (39 hospitals) were included. Extreme outliers were removed by excluding patients with a total hospital length of stay (LOS) more than 30 days.

### Hospitalization Costs

Trends in standardized unit costs represent our primary outcome. Standardized unit cost is not a direct measure of hospital dollar cost, but rather a measure of the volume of resources expended, expressed in dollar units.^14^ It is calculated based on hospital-specific median costs for individual services and was developed to serve as a surrogate for true resource use that is comparable across hospitals.^3^ Standardized unit costs in the PHIS database are already adjusted for the wage price index based on hospital location and were further adjusted to 2019 US dollars using the medical component of the consumer price index.^15^

### Patient Outcomes

Patient outcomes included LOS (total hospital and ICU), ICU admission rates, mechanical ventilation (invasive and noninvasive) rates, and mortality rates. Patients were classified as having received NIV if they had an *ICD-9* procedure code of 93.90 or 93.91, based on the high positive predictive value of this definition.^1,16^ It is known that some institutions code NIV for delivery of high-flow nasal cannula (HFNC); however, because there is no standard process for identifying HFNC using billing codes, we do not specifically report on this outcome. We used a previously published definition of invasive mechanical ventilation with a high positive predictive value, requiring a procedure or supply code for mechanical ventilation and a pharmacy charge for a neuromuscular blocking agent.^17^

### Patient Complexity and Illness Severity

Patient complexity included the proportion of patients with a complex chronic condition (CCC), whereas severity of illness included APR-DRG severity of illness (SOI) index scores. Presence of a CCC was determined by assignment of a diagnosis code in any of 10 published categories based on the pediatric CCCs classification system, version 2.^18^ Patients were assigned to APR-DRGs and received SOI index scores using 3M software.^19^

### Diagnostic Coding Practices

Diagnostic coding practices included the proportion of patients with bronchiolitis as their primary discharge diagnosis, diagnosis of respiratory failure, assignment to specific APR-DRGs, and the top 10 APR-DRGs assigned to patients with a diagnosis of bronchiolitis in any position. We were specifically interested in coding trends in the diagnosis of respiratory failure given its importance in determining APR-DRG classification. We were also interested in APR-DRG SOI index scores given their potential contribution to costs. The diagnosis of respiratory failure was identified by *ICD-9* diagnosis codes for acute respiratory failure (code 518.81), acute and chronic respiratory failure (code 518.84), and acute respiratory failure following trauma and surgery (code 518.51). We examined trends in APR-DRG codes for respiratory failure (code 133), bronchiolitis and respiratory syncytial virus pneumonia (code 138), and “other APR-DRG” for patients not assigned to those codes. Severity of illness index scores are specific within each APR-DRG, ranging from 1 to 4, with 4 denoting the sickest patients. Each unique combination of APR-DRG and SOI index score is assigned a specific service intensity weight.^20^ When used for reimbursement purposes, a hospital’s base rate is multiplied by the APR-DRG-SOI index score payment weight.

### Preplanned Subgroup Analyses

For our main analysis and sensitivity analyses, we performed preplanned subgroup analyses in which we examined nested subgroups as follows: (1) all patients hospitalized with bronchiolitis, (2) exclusion of patients with a CCC, (3) exclusion of patients who received mechanical ventilation or had a CCC, and (4) exclusion of patients with a CCC who received mechanical ventilation or who were admitted to the ICU. This stepwise narrowing of our cohort allowed us to systematically identify factors associated with the cost trends observed.

### Post Hoc Subgroup Analysis

We also performed a post hoc subgroup analysis in which we examined outcomes in the subgroup assigned to the respiratory failure APR-DRG. This analysis was performed after noting the marked increases in the assignment of this APR-DRG during our study period and sought to delineate whether the shift to this APR-DRG was due to increasing illness severity or coding intensity.

### Statistical Analysis

Cost and LOS were examined using quantile regression given the skewed distribution of these outcomes, whereas logistic regression was used to examine categorical outcomes. We examined calendar year as a categorical variable for our factor of interest, with models adjusted for age, sex, race, ethnicity, and insurance status. For race and ethnicity, we included a missing data indicator to avoid exclusion of patients for whom data were missing. Because the SOI index score was an outcome of interest, we did not adjust for differences in the SOI index score. Separate models incorporating year as a continuous factor were used as a test for trend. Statistical significance was set at 2-sided *P* < .001 to ensure the strength of the association given the large sample size. Between-hospital comparisons in the proportion of patients with a diagnosis of respiratory failure were adjusted for age, sex, race, ethnicity and insurance status using mixed-effects multilevel regression with hospital as a random intercept. Analyses were performed using Stata, version 15 (StataCorp).

## Results

A total of 385 883 bronchiolitis hospitalizations were included in the study; the patients had a mean (SD) age of 7.7 (6.4) months and included 227 309 of 385 883 boys (58.9%) and 253 870 of 385 883 publicly insured patients (65.8%) (Table). There was no change in the distribution of sex over time. Patients were increasingly likely to be classified in any race category except American Indian or “other” over time. The proportion of patients identifying as Hispanic and those with public insurance decreased over time. There was also a trend toward increasing mean age. The total number of bronchiolitis hospitalizations per year and the proportion of stays designated as observation stays in this cohort of children’s hospitals almost doubled during the study period.

**Table.** Demographic Characteristics and Outcomes for the Main Analysis of Patients With a Diagnosis of Bronchiolitis in Any Position

Demographic characteristic or outcome	2010	2012	2014	2016	2018	2019	*P* value for trend
Total bronchiolitis hospitalizations, No.	29 270	33 180	32 218	41 011	47 453	53 611	NA
Bronchiolitis in first discharge diagnosis position, % (95% CI)	45.7 (45.1-46.3)	45.2 (44.6-45.7)	39.9 (39.4-40.4)	37.2 (36.8-37.7)	34.8 (34.4-35.2)	33.9 (33.5-34.3)	<.001
Respiratory failure in first discharge diagnosis position, % (95% CI)	0.7 (0.6-0.8)	0.9 (0.8-1.0)	1.4 (1.3-1.6)	5.3 (5.1-5.5)	9.5 (9.2-9.8)	11.4 (11.2-11.7)	<.001
Observation status, % (95% CI)	15.2 (14.8-15.6)	22.7 (22.2-23.1)	25.1 (24.7-25.6)	25.4 (24.9-25.8)	24.5 (24.1-25.5)	25.1 (24.7-25.5)	<.001
Patient demographic characteristics							
CCC, No. (%)	4350 (14.9)	4993 (15.0)	5761 (17.9)	7878 (19.2)	8723 (18.4)	9600 (17.9)	<.001
Male, No. (%)	17 016 (58.1)	19 422 (58.5)	19 142 (59.4)	24 199 (59.0)	28 059 (59.1)	31 566 (58.9)	.01
Age, mean (SD), mo	6.5 (6.1)	6.8 (6.2)	7.5 (6.3)	7.7 (6.4)	8.1 (6.5)	8.2 (6.5)	<.001
Race, No. (%)							
American Indian	303 (1.0)	372 (1.1)	205 (0.6)	228 (0.6)	256 (0.5)	270 (0.5)	<.001
Asian	641 (2.2)	701 (2.1)	856 (2.7)	1159 (2.8)	1428 (3.1)	1699 (3.2)	<.001
Black	5427 (18.5)	6590 (19.9)	6954 (21.6)	8817 (21.5)	10 423 (22.0)	11 543 (21.5)	<.001
Pacific Islander	130 (0.4)	223 (0.7)	247 (0.8)	277 (0.7)	375 (0.8)	453 (0.8)	<.001
White	16 171 (55.2)	17 767(53.5)	17 490 (54.3)	22 233 (54.2)	26 311 (55.4)	29 892 (55.8)	<.001
Other	5433 (18.6)	5071 (15.3)	5176 (16.1)	6079 (14.8)	6164 (13.0)	7022 (13.1)	<.001
Missing	1165 (4.0)	2456 (7.4)	1290 (4.0)	2218 (5.4)	2496 (5.3)	2732 (5.1)	.014
Ethnicity, No. (%)							
Hispanic	8056 (27.5)	7909 (23.8)	7985 (24.8)	9417 (23.0)	10 321 (21.7)	12 549 (23.4)	<.001
Not Hispanic	16 484 (56.3)	21 452 (64.7)	21 251 (66.0)	28 352 (69.1)	33 936 (71.5)	37 772 (70.5)	<.001
Missing	4730 (16.2)	3819 (11.5)	2982 (9.3)	3242 (7.9)	3196 (6.7)	3290 (6.1)	<.001
Insurance, No. (%)							
Public	19 301 (65.9)	22 541 (67.9)	21 916 (68.0)	26 836 (65.4)	30 477 (64.2)	33 043 (61.6)	<.001
Private	9911 (33.9)	10 109 (30.5)	10 172 (31.6)	13 749 (33.5)	16 590 (35.0)	19 392 (36.2)	<.001
Missing	58 (0.2)	530 (1.6)	130 (0.4)	426 (1.0)	386 (0.8)	1176 (2.2)	<.001
APR-DRG							
Bronchiolitis^a^	78.1 (77.6-78.6)	79.8 (79.4-80.3)	75.5 (75.1-76.0)	69.4 (68.9-69.8)	65.3 (64.8-65.7)	59.3 (58.9-59.8	<.001
Respiratory failure^a^	1.2 (1.1-1.3)	1.4 (1.2-1.5)	3.5 (3.3-3.7)	9.2 (8.9-9.5)	14.6 (14.2-14.9)	21.6 (21.2-21.9)	<.001
Other^a^	20.8 (20.3-21.2)	18.9 (18.4-19.3)	20.9 (20.5-21.3)	21.4 (21.0-21.8)	20.2 (19.9-20.6)	19.0 (18.7-19.4)	.528
Total hospital length of stay, median (95% CI), d^a^	2.0 (2.0-2.0)	2.0 (2.0-2.0)	2.0 (2.0-2.0)	2.0 (2.0-2.0)	2.0 (2.0-2.0)	2.0 (2.0-2.0)	>.99
ICU admission^a^	12.4 (12.1-12.8)	15.4 (15.0-15.8)	17.9 (17.5-18.3)	22.1 (21.7-22.5)	25.4 (25.1-25.9)	26.9 (26.5-27.2)	<.001
ICU length of stay, median (95% CI), d^a^	3.1 (3.1-3.2)	2.7 (2.7-2.8)	2.7 (2.7-2.8	2.7 (2.6-2.7)	2.5 (2.5-2.5)	2.4 (2.4-2.5)	<.001
Mechanical ventilation^a^							
Invasive	3.8 (3.6-4.0)	3.7 (3.5-3.8)	3.7 (3.5-3.9)	4.3 (4.1-4.4)	4.0 (3.9-4.2)	3.5 (3.4-3.7)	.002
Noninvasive	1.6 (1.5-1.8)	2.6 (2.4-2.8)	4.0 (3.8-4.2)	6.1 (5.9-6.4)	8.8 (8.6-9.1)	9.8 (9.6-10.1)	<.001
Mortality^a^	0.1 (0.1-0.1)	0.1 (0.1-0.1)	0.1 (0.1-0.1)	0.1 (0.1-0.2)	0.1 (0.1-0.2)	0.1 (0.1-0.1)	.19

Abbreviations: APR-DRG, All Patient Refined Diagnosis Related Group; CCC, complex chronic condition; ICU, intensive care unit.

^a^ Outcomes were adjusted for age, sex, race, ethnicity, and insurance status.

### Trends in Hospitalization Costs

**Figure 1.**
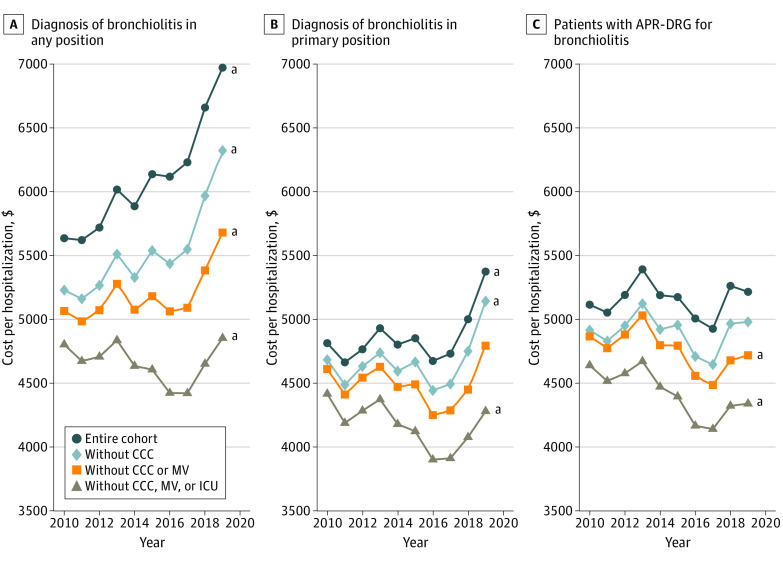
Standardized Unit Costs for Preplanned Nested Subgroup Analysis A, Patients with a diagnosis of bronchiolitis in any position. B, Patients with a diagnosis of bronchiolitis in the primary position. C, Patients with assignment of the All Patient Refined Diagnosis Related Group (APR-DRG) for bronchiolitis. CCC indicates complex chronic condition; ICU, intensive care unit; and MV, mechanical ventilation. ^a^Statistical significance for the trend from 2010 to 2019.

Among all patients hospitalized with bronchiolitis, the median standardized unit cost per hospitalization increased significantly during the study period (from $5636 [95% CI, $5558-$5714] in 2010 to $6973 [95% CI, $6915-$7030] in 2019; *P* < .001 for trend). However, costs for patients without a CCC or mechanical ventilation who received care outside the intensive care unit did not change in an economically significant manner (from $4803 [95% CI, $4752-$4853] in 2010 to $4853 [95% CI, $4811-$4895] in 2019; *P* < .001 for trend). Preplanned nested subgroup analyses of patients, designed to identify factors associated with increasing cost, are shown in Figure 1, which provides trends for patients with bronchiolitis in any diagnosis position (Figure 1A), patients with a primary diagnosis of bronchiolitis (Figure 1B), and patients assigned to the bronchiolitis APR-DRG (Figure 1C).

Figure 1A demonstrates similar trends of increasing costs in the broadest cohort regardless of patient-level factors. When cost was analyzed by including only patients with bronchiolitis in the primary discharge diagnosis position (Figure 1B), modest cost increases were noted for the entire cohort and patients without a CCC, while costs decreased for patients who received care outside the ICU. When cost was analyzed by including only patients assigned to the APR-DRG for bronchiolitis (Figure 1C), the only statistically significant trends were decreased costs for patients without a CCC who did not receive mechanical ventilation and for patients treated outside the ICU.

### Trends in Patient Outcomes

For the entire cohort, the median total hospital LOS remained stable at 2.0 days (95% CI, 2.0-2.0 days; *P* > .99 for trend) (Table). The proportion of patients treated in the ICU more than doubled from 12.4% (95% CI, 12.1%-12.8%) to 26.9% (95% CI, 26.5%-27.2%; *P* < .001 for trend), while the median ICU LOS decreased by 0.7 days (95% CI, 3.1-3.2; *P* < .001 for trend). The use of mechanical ventilation increased from 1.6% (95% CI, 1.5%-1.8%) to 9.8% (95% CI, 9.6%-10.1%; *P* < .001 for trend), associated primarily with a more than 6-fold increase in the proportion of patients who received NIV, as the use of invasive mechanical ventilation remained stable. Mortality remained rare and unchanged at 0.1%. Findings for the preplanned subgroup analyses (eTable 1 in the Supplement) were similar to the primary analyses.

### Trends in CCCs and SOI

A total of 67 505 hospitalizations (17.5%) were for patients with a CCC. When examining trends in APR-DRG and SOI index score together, the largest shifts were away from bronchiolitis APR-DRG SOI index scores of 1 and 2 (combined 72.4% in 2010 compared with 44.3% in 2019) and toward bronchiolitis APR-DRG SOI index score of 3 and respiratory failure APR-DRG SOI index score of 2 (combined 5.3% in 2010 compared with 30.9% in 2019) (eTable 1 in the Supplement).

**Figure 2.**
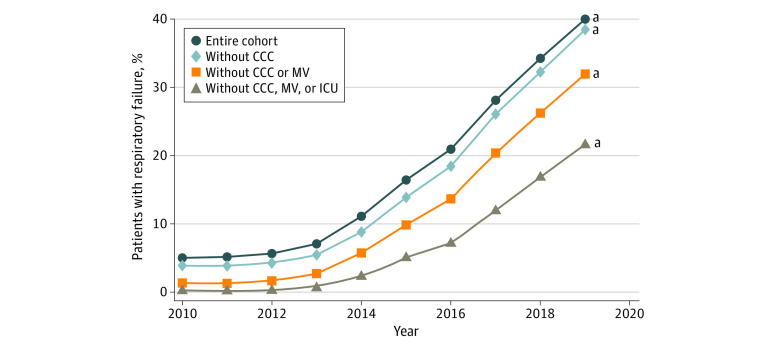
Adjusted Proportion of Patients With a Diagnosis of Respiratory Failure CCC indicates complex chronic condition; ICU, intensive care unit; and MV, mechanical ventilation. ^a^Statistical significance for the trend from 2010 to 2019.

**Figure 3.**
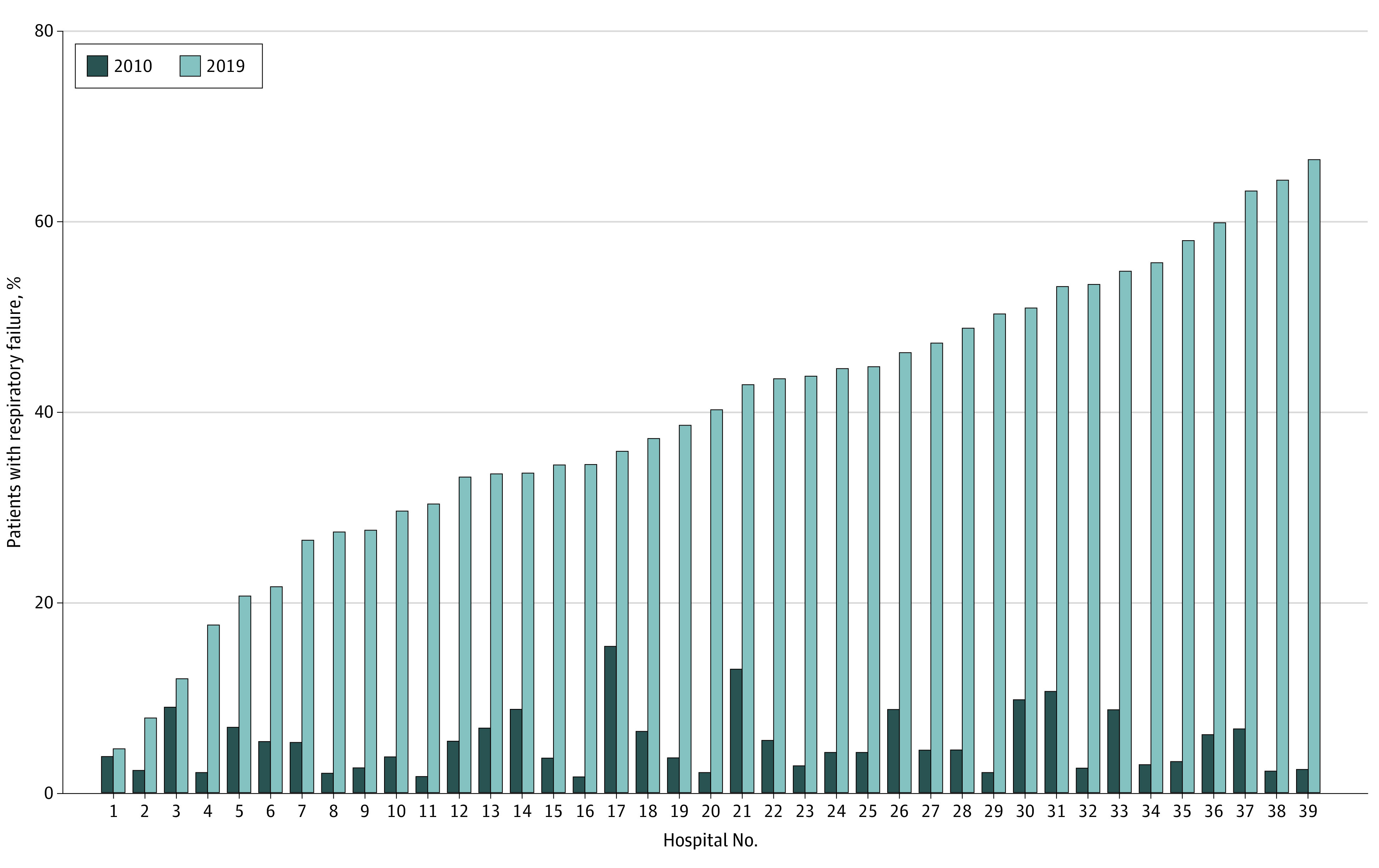
Adjusted Proportion of Patients With a Diagnosis of Respiratory Failure in 2010 and 2019 at Each Included Hospital

**Figure 4.**
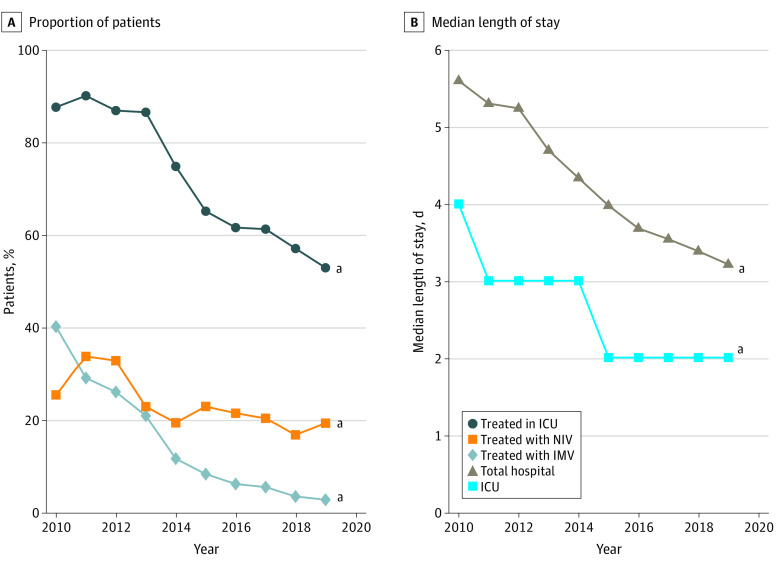
Adjusted Outcomes for Patients With an All Patient Refined Diagnosis Related Group Assignment for Respiratory Failure A, Proportion of patients. B, Median length of stay. ICU indicates intensive care unit; IMV, invasive mechanical ventilation; and NIV, noninvasive mechanical ventilation. ^a^Statistical significance for the trend from 2010 to 2019.

The diagnosis of respiratory failure in any position increased markedly from 5.2% (95% CI, 4.8%-5.3%) in 2010 to 40.0% (95% CI, 39.6%-40.4%) in 2019, a trend that was observed across all patient subgroups. This included the lowest-acuity group (those without a CCC, not receiving mechanical ventilation, or not receiving care in the ICU), of whom nearly one-fourth received a diagnosis of respiratory failure in 2019 (Figure 2). This trend was also associated with increasing between-hospital variation in the diagnosis of respiratory failure between 2010 and 2019 (Figure 3). Post hoc subgroup analysis including only patients assigned to the respiratory failure APR-DRG revealed notable differences in patient outcomes in 2019 compared with 2010, with this group of patients experiencing significantly shorter total and ICU LOS, decreased ICU admission and mechanical ventilation use, and lower mortality rates (Figure 4).

### Trends in Diagnostic Coding Practices

The proportion of patients with a discharge diagnosis of bronchiolitis in the primary position decreased from 45.7% (95% CI, 45.1%-46.3%) in 2010 to 33.9% (95% CI, 33.5%-34.3%; *P* < .001 for trend) in 2019 (Table), whereas a diagnosis of respiratory failure in the primary position increased from 0.7% (95% CI, 0.6%-0.8%) in 2010 to 11.4% (95% CI, 11.2%-11.7%; *P* < .001 for trend) in 2019. Similar patterns were observed in APR-DRG assignment, with the proportion of patients assigned to the bronchiolitis APR-DRG decreasing by 18.8% (from 78.1% [95% CI, 77.6%-78.6%] in 2010 to 59.3% [95% CI, 58.9%-59.8%] in 2019), while the proportion of patients assigned to the respiratory failure APR-DRG increased by 20.4% (from 1.2% [95% CI, 1.1%-1.3%] in 2010 to 21.6% [95% CI, 21.2%-21.9%] in 2019; *P* < .001 for trend) (Table). The proportion of patients assigned to an APR-DRG for something other than bronchiolitis or respiratory failure remained stable, suggesting direct substitution of the respiratory failure APR-DRG for the bronchiolitis APR-DRG. The top 10 APR-DRG assignments for patients with bronchiolitis in any diagnosis position show that most patients were assigned to the APR-DRG for “bronchiolitis and RSV [respiratory syncytial virus] pneumonia” or “respiratory failure” (cumulative proportion, 80%; eTable 2 in the Supplement).

## Discussion

Data from this cohort of children’s hospitals revealed increasing costs per hospitalization for patients with bronchiolitis from 2010 to 2019. Costs per hospitalization were higher when including patients with CCCs, although the rate of increase was similar regardless of medical complexity. Furthermore, costs increased among patients without a CCC or mechanical ventilation, suggesting that complexity of condition and mechanical ventilation are unlikely explanations for the observed trends. The only group that did not see increased costs across the period studied was patients who received care outside the ICU, suggesting that increasing costs for bronchiolitis are associated with higher ICU use.

The proportion of patients treated in the ICU more than doubled, while the use of NIV increased to an even greater extent, suggesting that increasing illness severity among hospitalized children may be responsible for increasing resource use. However, the decision to use such resources is often based on clinical judgement. Therefore, absent worsening patient outcomes as judged by stable total hospital LOS, invasive mechanical ventilation rates, and mortality, it is unclear whether the trends we observed in resource use are entirely patient driven. A shorter mean ICU LOS suggests that the threshold for ICU use may have decreased. If this is the case, the mean illness severity of patients who received care outside the ICU would have decreased over time, which is consistent with the cost trends observed in this subgroup. We also observed important trends in diagnostic coding, most notably, increased use of the code for respiratory failure. This trend was associated with a parallel shift of patients being assigned to the respiratory failure APR-DRG and to higher SOI index scores within the bronchiolitis APR-DRG. These shifts would seem to imply higher patient acuity; however, decreasing total hospital and ICU LOSs, ICU use, mechanical ventilation rates, and mortality for the respiratory failure APR-DRG cohort do not support this conclusion.

It is not clear why resource use and apparent illness severity are increasing despite stable or improving patient outcomes. One possibility is that the widespread adoption of HFNC may be associated with observed trends as ward-based HFNC protocols have been associated with increased ICU use.^21,22,23,24^ Its use may also justify the diagnosis of respiratory failure and may be associated with increased SOI index scores. As such, increasing HFNC use could explain both increasing costs and apparent increasing patient acuity. Unfortunately, the PHIS database is unable to reliably identify HFNC use.^22^ Another potential explanation for our observed coding trends is that an increased emphasis on accurate documentation and coding in hospitals could be associated with shifts in the APR-DRG SOI index score over time that are not dependent on patient-level factors.^12^ States assign service intensity weights for each APR-DRG SOI index score category, which are used for hospital case-mix adjustment to adjust quality reporting and reimbursement, providing a strong motivation for attention to diagnostic coding. As an example, an APR-DRG SOI index score of 2 for bronchiolitis carries a state-specific service intensity weight of 0.6303, whereas an APR-DRG SOI index score of 2 for respiratory failure carries a state-specific service intensity weight of 0.9234, leading directly to higher facility reimbursement.^20^ Given the discrepancies in the formal definition of respiratory failure and how it is commonly used in clinical settings, we cannot determine from our data whether undercoding predominated early in our study period or whether overcoding predominated in later years.

Our findings have implications for future studies on bronchiolitis, particularly when studying trends over time. Owing to significant shifts in coding practices, inclusion of only patients with a primary diagnosis of bronchiolitis or those who have been assigned to an APR-DRG for bronchiolitis will exclude a increasing proportion of patients hospitalized with bronchiolitis and may lead to biased results. This is particularly true if patients with a diagnosis of respiratory failure are excluded because this diagnosis seems to be replacing bronchiolitis as the primary diagnosis for some patients with bronchiolitis. Furthermore, if the patient SOI index score is changing because of diagnostic coding practices rather than patient-level factors, using it in models as a covariate is likely to be associated with an inaccurate adjustment for the SOI index score. Such coding bias has been demonstrated among the adult Medicare population, and pediatric health services researchers should be cautious in assuming that diagnoses obtained from claims data are accurate and useful in reducing confounding.^25,26,27^

### Limitations

Our study has some limitations. Research using administrative data is subject to multiple limitations, and some degree of error in patient classification is undoubtedly present. The PHIS database lacks detailed clinical information; thus, our ability to evaluate true illness severity is limited. Furthermore, our primary outcome measure represents an approximation of costs, rather than actual health care costs or spending, and statistically significant differences may not be economically meaningful. Finally, data from the PHIS database are limited to children’s hospitals, and shifts in the care of pediatric patients from community hospitals to children’s hospitals could affect our observed trends. However, it is unlikely that consolidation of pediatric services has led to a sicker population being treated in children’s hospitals, which is supported by the observed stability in our more objective outcomes, such as LOS, use of invasive mechanical ventilation, and mortality.

## Conclusions

This cross-sectional study found that hospitalized children with bronchiolitis are receiving more costly and intensive care without objective evidence of increasing severity of illness. Findings suggest that increased coding intensity associated with ICU and NIV use may complicate efforts to study trends in use of health care resources using administrative data. Future research on trends in bronchiolitis should account for changes in the use of diagnostic coding.

## Notes

Supplement. **eTable 1.** Outcomes for the Preplanned Nested Subanalyses for Patients With Bronchiolitis in any Diagnosis Position **eTable 2.** The Number of Patients, Proportion of Patients and Cumulative Proportion of Patients for each of the 10 most Common APR-DRGs for Patients With Bronchiolitis in any Diagnosis Position
