# Supplementary material for: Trends in Hospital Costs and Levels of Services Provided for Children With Bronchiolitis Treated in Children’s Hospitals
Source: JAMA Netw Open. 2021 Oct 26;4(10):e2129920. doi: 10.1001/jamanetworkopen.2021.29920 (PMC8548950; doi:10.1001/jamanetworkopen.2021.29920)
Supplement: Supplement. — eTable 1. Outcomes for the Preplanned Nested Subanalyses for Patients With Bronchiolitis in any Diagnosis Position eTable 2. The Number of Patients, Proportion of Patients and Cumulative Proportion of Patients for each of the 10 most Common APR-DRGs for Patients With Bronchiolitis in any Diagnosis Position [file jamanetwopen-e2129920-s001.pdf]

Supplementary Online Content

Willer RJ, Coon ER, Harrison WN, Ralston SL. Trends in hospital costs and levels of services provided for children with bronchiolitis treated in children's hospitals. *JAMA Netw Open.* 2021;4(10):e2129920. doi:10.1001/jamanetworkopen.2021.29920

eTable 1. Outcomes for the Preplanned Nested Subanalyses for Patients With Bronchiolitis in any Diagnosis Position

eTable 2. The Number of Patients, Proportion of Patients and Cumulative Proportion of Patients for each of the 10 most Common APR-DRGs for Patients With Bronchiolitis in any Diagnosis Position

This supplementary material has been provided by the authors to give readers additional information about their work.

© 2021 Willer RJ et al. *JAMA Network Open.*

eTable 1. Outcomes for the Preplanned Nested Subanalyses for Patients With Bronchiolitis in any Diagnosis Position. All outcomes were adjusted for age, sex, race, ethnicity and insurance status.

**Outcomes**	**2010**	**2012**	**2014**	**2016**	**2018**	**2019**	***P* value for trend**
Bronchiolitis APR-DRG (%)							
Without CCC	81.9 (81.4-82.4)	83.6 (83.2-84.1)	80.2 (79.7-80.6)	74.6 (74.2-75.1)	69.7 (69.3-70.2)	63.3 (62.9-63.8	<.001
Without CCC or MV	83.9 (83.5-84.5)	85.8 (85.4-86.2)	82.2 (81.8-82.7)	78.0 (77.5-78.4)	72.7 (72.2-73.1)	67.1 (66.6-67.6	<.001
Without CCC, MV, or ICU	84.7 (84.3-85.2)	86.6 (86.1-87.0)	84.2 (83.7-84.6)	81.5 (81.0-82.0)	77.6 (77.2-78.1)	72.8 (72.4-73.3)	<.001
Respiratory Failure APR-DRG (%)							
Without CCC	1.1 (0.9-1.2)	1.2 (1.1-1.3)	3.3 (3.1-3.5)	9.3 (8.9-9.6)	15.2 (14.8-15.5)	22.4 (22.0-22.7)	<.001
Without CCC or MV	0.4 (0.3-0.5)	0.6 (0.5-0.7)	2.5 (2.3-2.7)	7.5 (7.2-7.8)	13.6 (13.3-14.0)	19.7 (19.3-20.1)	<.001
Without CCC, MV, or ICU	0.1 (0.1-0.1)	0.1 (0.1-0.2)	1.0 (0.9-1.1)	4.3 (4.1-4.6)	8.4 (8.1-8.7)	13.7 (13.4-14.1)	<.001
APR-DRG SOI, % (95% CI)							
Bronchiolitis APR-DRG SOI 1	43.7 (43.2-44.2)	42.9 (42.4-43.3)	33.8 (33.4-34.3)	36.6 (36.2-37.0)	29.6 (29.2-29.9)	27.7 (27.3-28.0)	<.001
Bronchiolitis APR-DRG SOI 2	28.6 (28.2-29.1)	30.1 (29.6-30.6)	31.7 (31.2-32.2)	22.2 (21.9-22.6)	19.0 (18.6-19.3)	16.7 (16.4-17.0)	<.001
Bronchiolitis APR-DRG SOI 3	4.9 (4.7-5.1)	6.0 (5.8- 6.3)	8.7 (8.4-9.0)	9.0 (8.8-9.3)	15.2 (14.8-15.5)	13.8 (13.5-14.1)	<.001
Bronchiolitis APR-DRG SOI 4	1.0 (0.9-1.1)	0.9 (0.8-1.0)	1.2 (1.1-1.3)	1.5 (1.4-1.6)	1.7 (1.6-1.8)	1.3 (1.2-1.4)	<.001
Respiratory Failure APR-DRG SOI 1	0.0 (0.0-0.0)	0.0 (0.0-0.0)	0.0 (0.0-0.0)	0.0 (0.0-0.0)	0.0 (0.0-0.0)	0.0 (0.0-0.0)	.689
Respiratory Failure APR-DRG SOI 2	0.4 (0.3-0.5)	0.6 (0.5-0.7)	2.0 (1.9-2.1)	6.5 (6.3-6.6)	11.0 (10.8-11.1)	17.1 (16.9-17.2)	<.001

© 2021 Willer RJ et al. *JAMA Network Open.*

Respiratory Failure APR-DRG SOI 3	0.4 (0.3-0.5)	0.5 (0.4-0.5)	1.0 (0.9-1.1)	2.0 (1.9-2.1)	2.9 (2.7-3.0)	3.7 (3.5-3.8)	<.001
Respiratory Failure APR-DRG SOI 4	0.4 (0.3-0.4)	0.3 (0.2-0.3)	0.5 (0.4-0.5)	0.7 (0.6-0.8)	0.7 (0.6-0.8)	0.9 (0.8-0.9)	<.001
Other APR-DRG (%)	20.8 (20.3-21.2)	18.9 (18.4-19.3)	20.9 (20.5-21.3)	21.4 (21.0-21.8)	20.2 (19.9-20.6)	19.0 (18.7-19.4)	.528
Hospital Length of Stay, days (median)							
Without CCC	2.0	2.0	2.0	2.0	2.0	2.0	>.99
Without CCC or MV	2.0	2.0	2.0	2.0	2.0	2.0	>.99
Without CCC, MV, or ICU	2.0	2.0	2.0	2.0	2.0	2.0	>.99
ICU admission (%)							
Without CCC	10.0 (9.6-10.4)	12.9 (12.5-13.3)	14.4 (14.0-14.8)	18.9 (18.5-19.3)	22.5 (22.0-22.9)	24.1 (23.7-24.5)	<.001
Without CCC or MV	6.9 (6.5-7.2)	9.7 (9.4-10.1)	10.7 (10.3-11.1)	13.8 (13.4-14.2)	15.4 (15.1-15.8)	16.7 (16.3-17.1)	<.001
ICU Length of Stay, days (median)							
Without CCC	2.7 (2.6-2.8)	2.6 (2.5-2.7)	2.5 (2.4-2.6)	2.5 (2.4-2.5)	2.3 (2.2-2.3)	2.2 (2.2-2.3)	<.001
Without CCC or MV	2.0	2.0	2.0	2.0	2.0	2.0	>.99
Mechanical Ventilation (%)							
Invasive							
Without CCC	2.6 (2.4-2.8)	2.5 (2.4-2.7)	2.3 (2.2-2.5)	2.7 (2.5-2.9)	2.6 (2.4-2.7)	2.3 (2.1-2.4)	.916
Non-Invasive							
Without CCC	1.2 (1.1-1.4)	2.0 (1.8-2.2)	3.3 (3.1-3.5)	5.5 (5.2-5.7)	8.1 (7.9-8.4)	9.1 (8.8-9.4)	<.001
Mortality (%)							
Without CCC	0.0 (0.0-0.0)	0.0 (0.0-0.0)	0.0 (0.0-0.0)	0.0 (0.0-0.0)	0.0 (0.0-0.0)	0.0 (0.0-0.0)	.564
Without CCC or MV	0.0 (0.0-0.0)	0.0 (0.0-0.0)	0.0 (0.0-0.0)	0.0 (0.0-0.0)	0.0 (0.0-0.0)	0.0 (0.0-0.0)	.558
Without CCC, MV, or ICU	0.0 (0.0-0.0)	0.0 (0.0-0.0)	0.0 (0.0-0.0)	0.0 (0.0-0.0)	0.0 (0.0-0.0)	0.0 (0.0-0.0)	.461

© 2021 Willer RJ et al. *JAMA Network Open.*

eTable 2. The Number of Patients, Proportion of Patients and Cumulative Proportion of Patients for each of the 10 most Common APR-DRGs for Patients With Bronchiolitis in any Diagnosis Position

APR-DRG	APR-DRG Description	Number of Patients	Proportion of Patients	Cumulative Proportion of Patients
138	Bronchiolitis and RSV pneumonia	274,138	71.04	71.04
133	Respiratory Failure	33,749	8.75	79.79
139	Other Pneumonia	15,487	4.01	83.8
132	BPD & other chronic respiratory diseases arising in perinatal period	8,546	2.21	86.02
130	Respiratory system diagnosis with ventilator support 96+ hours	7,734	2	88.02
141	Asthma	6,551	1.7	89.72
113	Infections of upper respiratory tract	4,011	1.04	90.76
422	Hypovolemia & related electrolyte disorders	3,400	0.88	91.64
144	Respiratory signs, symptoms & minor diagnoses	3,298	0.85	92.49
724	Other infectious & parasitic diseases	3,212	0.83	93.33

© 2021 Willer RJ et al. *JAMA Network Open.*
